# Liquisolid Systems to Improve the Dissolution of Furosemide

**DOI:** 10.3797/scipharm.0912-23

**Published:** 2010-04-23

**Authors:** Babatunde Akinlade, Amal A. Elkordy, Ebtessam A. Essa, Sahar Elhagar

**Affiliations:** 1 Faculty of Applied Sciences, Department of Pharmacy, Health and Well-being, University of Sunderland, Sunderland, SR1 3SD, United Kingdom; 2 Faculty of Pharmacy, Tanta University, Tanta, Egypt

**Keywords:** Liquisolid compacts, Furosemide, Dissolution, Friability, Synperonic® PE/L 81

## Abstract

A liquisolid system has the ability to improve the dissolution properties of poorly water soluble drugs. Liquisolid compacts are flowing and compactable powdered forms of liquid medications. The aim of this study was to enhance the in vitro dissolution properties of the practically water insoluble loop diuretic furosemide, by utilising liquisolid technique. Several liquisolid tablets were prepared using microcrystalline cellulose (Avicel® pH-101) and fumed silica (Cab-O-Sil® M-5) as the carrier and coating materials, respectively. Polyoxy-ethylene-polyoxypropylene-polyoxyethylene block copolymer (Synperonic® PE/L 81); 1,2,3-propanetriol, homopolymer, (9*Z*)-9-octadecenoate (Caprol® PGE-860) and polyethylene glycol 400 (PEG 400) were used as non- volatile water-miscible liquid vehicles. The liquid loading factors for such liquid vehicles were calculated to obtain the optimum amounts of carrier and coating materials necessary to produce acceptable flowing and compactible powder admixtures viable to produce compacts. The ratio of carrier to coating material was kept constant in all formulations at 20 to 1. The formulated liquisolid tablets were evaluated for post compaction parameters such as weight variation, hardness, drug content uniformity, percentage friability and disintegration time. The in-vitro release characteristics of the drug from tablets formulated by direct compression (as reference) and liquisolid technique, were studied in two different dissolution media. Differential scanning calorimetry (DSC) and Fourier-Transform infrared spectroscopy (FT-IR) were performed. The results showed that all formulations exhibited higher percentage of drug dissolved in water (pH 6.4–6.6) compared to that at acidic medium (pH 1.2). Liquisolid compacts containing Synperonic® PE/L 81 demonstrated higher release rate at the different pH values. Formulations with PEG 400 displayed lower drug release rate, compared to conventional and liquisolid tablets. DSC and FT-IR indicated a possible interaction between furosemide and tablet excipients that could explain the dissolution results. Caprol® PGE-860, as a liquid vehicle, failed to produce furosemide liquisolid compacts.

## Introduction

The solubility of active ingredient(s) is a matter of concern to formulators. The poor dissolution rates of poorly water soluble drugs is still a substantial problem confronting drug development, such as hindering the development of parenteral products and limiting the bioavailability of oral products [1]. Poorly soluble drugs that are administered orally will generally exhibit slow dissolution rates and incomplete bioavailability due to poor wettability of those drugs. This indicates that drugs are not sufficiently wetted before reaching absorption site [2]. From a physicochemical point of view, it is well established that the inadequate dissolution of poorly water soluble drugs is the major reason for their poor and erratic bioavailability, since it is the rate determining step in the absorption of those drugs.

As large proportions of new drug candidates have poor aqueous solubility, various formulation strategies were reported to overcome such a problem. Among these techniques is complexation with cyclodextrins, solid dispersion, co-precipitation and recently, the technique of ‘liquisolid compacts’. Several studies have shown that the liquisolid technique is a promising method for promoting dissolution rate of poorly water soluble drugs [3–9]. In liquisolid compact, a liquid medication is converted into acceptably flowing and compactible powder forms. The term ‘liquid medication’ implies liquid lipophilic (oily) drug and solution or suspension of poorly water soluble drugs carried in suitable water miscible non-volatile liquid systems termed the liquid vehicle. By simple blending with suitable excipients ‘carrier and coating materials’, the liquid medication may be converted into a dry-looking, non-adherent, free flowing and readily compactible powder [10]. Since drug dissolution is often the rate limiting step in gastrointestinal absorption, the significant increase in wetting properties and surface area of drug particles available for dissolution from liquisolid compacts may be expected to display enhanced drug release characteristics and, consequently, improved oral bioavailability.

The technique of liquisolid compacts has been successfully employed to improve the in vitro release of poorly water soluble drugs such as carbamazepine [11], famotidine [12], piroxicam [5], indomethacin [6], hydrocortisone [3], naproxen [8] and prednisolone [10].

Furosemide, 4-chloro-2-[(furan-2-ylmethyl)amino]-5-sulfamoylbenzoic acid, is a drug with a diuretic action which acts at the renal level on the ascending limb of the loop of Henle, and is used in the treatment of oedema of pulmonary, cardiac or hepatic origin as well as in the treatment of hypertension and in the chronic treatment of cardiac infarction [13]. The major problem associated with the formulation and effectiveness of the furosemide is its variable oral absorption of about 11–90% [14] due to insufficient aqueous solubility at gastrointestinal pH, thus making solubility the rate-determining step in the gastric absorption of furosemide [15]. Therefore, furosemide establishes a good candidate for testing the potential of rapid-release liquisolid compacts

The aim of this study was to increase dissolution rate of furosemide using liquisolid technique. The drug was formulated into 20 mg liquisolid tablets using Synperonic® PE/L 81, Caprol® PGE-860 and PEG 400 as the water miscible non-volatile liquid vehicles. Theoretical model of liquisolid systems [4] was used to calculate the appropriate quantity of excipients (carrier and coating materials) for each liquid vehicle required to produce acceptably flowing and compactible powders. The liquisolid powder system which showed acceptable flowability was then compacted into tablets and the in vitro drug dissolution rates of liquisolid formulations were compared to that of conventional, directly compacted tablets. There is no single non-volatile liquid vehicle which is suitable for a variety of hydrophobic drugs in preparing liquisolid tablets. Propylene glycol, Tween 80 and polyethylene glycol 400 (PEG400) had been used as non-volatile liquid vehicles in the preparation of fast release liquisolid tablets with different drugs [4, 6, 7, 10, 14]; El-Gizawy [16] reported that polysorbate 80 shows better dissolution rate than propylene glycol and PEG400 when used with meloxicam. From the other hand, Spireas and Sadu [10]; Nokhodchi et al. [6] reported that liquisolid tablets containing propylene glycol show higher dissolution rate than those containing PEG400 or polysorbate 80. Accordingly in this study besides PEG400, Caprol® PGE-860 and Synperonic® PE/L 81, which have not, to the best of our knowledge, been studied before to enhance dissolution of furosemide, were used as non-volatile liquid vehicles in the liquisolid systems containing furosemide to enhance its dissolution.

## Results and Discussion

### Solubility measurement

The solubility of furosemide in the non-volatile liquid vehicles, Synperonic® PE/L 81, Caprol® PGE 860, PEG 400 as well as water was determined. The results showed greater solubility of the drug in all vehicles in comparison to water. The amounts (μg/ml) of furosemide dissolved were 4.47 ±1.2, 34.6 ±2.6, 42.3 ±3.1 and 46.0 ±2.9 in water, PEG 400, Synperonic® PE/L 81 and Caprol® PGE 860, respectively. The higher solubility of the drug in Caprol® PGE 860 compared with other liquid vehicles may be due to the longer non-polar chain of Caprol® PGE 860. The longer non-polar part is thought to reflect hydrophobic interactions of the drug with the liquid vehicle molecule [16].

### Flowable liquid retention potentials (Φ-values) and liquid load factors (L_f_) for Avicel® PH 101 and fumed silica

**Fig. 1.**
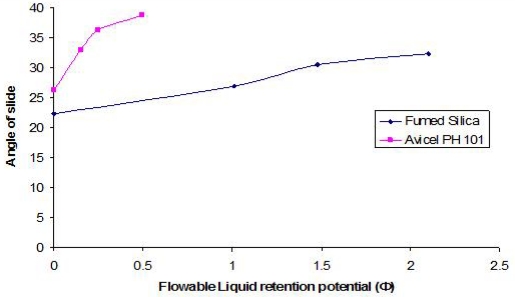
Relationship between the angle of slide of Avicel® PH 101 and fumed silica with Synperonic® PE/L 81 as a liquid vehicle

**Fig. 2.**
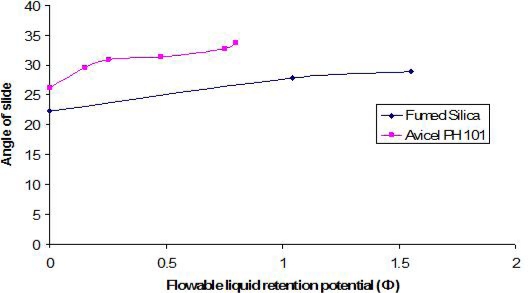
Relationship between the angle of slide of Avicel® PH 101 and fumed silica in Caprol® PGE 860 as a liquid vehicle

The optimum angle of slide for both carrier and coating materials were determined in order to calculate the optimum liquid loading factor (L_f_) for each of them in Syperonic® PE/L 81 and Caprol® PGE 860 (as discussed in Experimental Section, see below). The angle of slides for the carrier and coating materials alone and in the presence of different concentrations of the liquid vehicles are graphically presented in Figures (1 and 2). As a general trend, the angle of slide for both carrier and coating materials increased as the flowable liquid retention potentials increases.

The flowable liquid retention potentials (ΦCA-value) for Avicel® PH 101 in Synperonic® PE/L 81 and Caprol® PGE 860 corresponding to an angle of slide of 33º were approximately 0.164 and 0.76, respectively.

For fumed silica, the flowable liquid retention potential (ΦCO-value) in Synperonic® PE/L 81 corresponding to an angle of slide of 33° was equal to approximately 2.20. However, the angle of slide of silica in Caprol® PGE 860 (28.98°) was below 33° (Figure 2). Furthermore, any increase in ΦCO-value more than 1.55 resulted in decreasing the angle of slide. As a result, the angle closet to 33° was chosen “which corresponded to a resultant ΦCO-value of 1.55”. Flowable liquid retention potential values for Avicel® PH 101 and fumed silica in PEG 400 were taken from literature [4]. The Liquid load factors (L_f_) were then calculated using Equation (1). For each non-volatile liquid vehicle, the corresponding L_f_ value is presented in Table (2).

### Percentage compressibility studies of different powder systems

Powder flow is a critical character that might affect uniformity of tablet weight. Therefore, the flow properties of the powder admixture of conventional and liquisolid formulations were determined in order to ensure that the amount of carrier and coating materials are enough to maintain acceptable flow and compaction properties. The Carr’s index (CI%) or percentage compressibility were calculated for conventional and liquisolid powder admixtures. Previous studies have shown that in preparation of liquisolid tablets, it is difficult to prepare formulations with good flowability and compactibility when liquid loading factor is significantly above 0.25 [5]. The liquid load factors for PEG 400, Synperonic® PE/L 81 and Caprol® PGE 860 were 0.168, 0.274 and 0.8375, respectively (Table 2). The liquid load factor for Caprol® PGE 860 was approximately 3 times greater than 0.25. The prepared powder admixtures LS-5 and LS-6 (with Caprol® PGE 860) showed poor flowability and were not compactible. In addition, those powder admixtures were in the form of aggregates with some segregation noticed during powder mixing, whilst the powders for the remaining liquisolid formulations appeared fine (LS-1 to LS-4). Such behaviour of LS-5 and LS-6 powder systems could possibly coincides with high liquid load factor of Caprol® PGE 860, and indicates poor flowability. Other explanation for this failure could be based on the viscosity of the liquid vehicle as it was stated that the viscosity of the vehicles affects the flowability of powders [17].

The dynamic viscosity of PEG 400, Synperonic® PE/L 81 and Caprol® PGE 860 were measured (See Section 2.4) and found to be 111.3, 454, and 5276 mPas, respectively. Therefore, it could also be suggested that the poor flowability of powder prepared using Caprol® PGE 860 is due to its high viscosity which may hindered powder mixing. Therefore, liquisolid formulations LS-5 and LS-6 were considered as unsuccessful and will not, therefore, be considered further in our discussion.

For the conventional tablets, and the liquisolid formulations, CI% values were 19.6 ±2.6%, 4.8 ±0.64%, 18.6 ±2.1%, 13.6 ±1.5%, and 20 ±1.7% for CDT, LS-1, LS-2, LS-3 and LS-4, respectively. It was stated that powder showing compressibility index between 5 and 15% is considered to have excellent flowability, whilst CI% values between 12 and 16% is of good flowability and up to 21 is fair to passable [18]. Therefore, we can say that CI% values of all liquisolid powder admixtures were less than (P < 0.05) that of conventional powder system, except formula LS-4 that showed a similarity (P > 0.05) to the conventional type. Therefore, such decrease in compressibility index would indicate substantial improvement in flow and packing ability of the powder mass of liquisolid formulations in comparison to the conventional type. The reason for flow improvement of liquisolid formulations can be attributed to use of the appropriate amounts of Avicel® PH 101 and fumed silica as calculated by applying Equations 1–3, accordingly non-adherent and free flowing powders were produced. The overall results reflect that all tested powder systems have acceptable flowability that would suggest a uniform and reproducible die filling resulting in tablets of uniform weights upon compression.

### Evaluation of furosemide liquisolid (LS-1 to LS-4) and conventional (DCT) tablets

Table 3 shows the results of tests performed to evaluate the physical properties of the prepared tablets according to British Pharmacopoeia [19].

It is clear that conventional (CDT) and liquisolid tablets complied with the required specifications and standard regarding drug content uniformity. Liquisolid tablets complied with British Pharmacopeia friability test as the friability was less than 1% and there were no broken tablets, whilst conventional tablets (Table 3) exhibited percentage friability more than 1% (not comply with the British Pharmacopeia specification). Therefore, conventional tablets could not withstand fracturing and attrition during normal handling and transport.

For the disintegration time results, CDT, LS-1 and LS-2 showed very short disintegration time (few seconds), while that for LS-3 and LS-4 was prolonged. The rapid disintegration of CDT might be due to tablet friability as it showed the highest percent friability and less cohesion compared to all other formulations (Table 3). For LS-1 and LS-2, though showed acceptable friability and tablet cohesion, they displayed rapid disintegration. This could be due to the surface activity of Synperonic® PE/L 81 that would facilitate tablet wetting by reducing the interfacial tension. On the other hand, PEG 400, with its two terminal hydroxyl groups, may strengthen the binding of the tablet by the formation of hydrogen bonding with Avicel® PH 101 (as will be confirmed by FT-IR data), thus conferring cohesion to the compacts as reflected by the hardness results (Tab 3). This might resulted in prolongation of the tablet disintegration times that would, therefore, affect tablet dissolution [12].

### In vitro dissolution studies

**Fig. 3.**
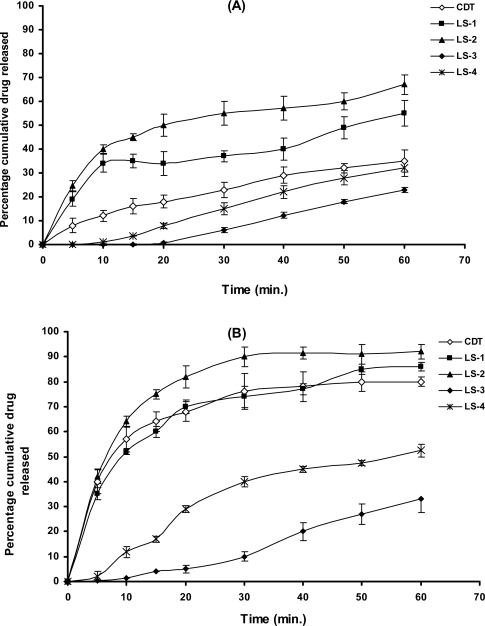
Dissolution profiles of furosemide from conventional and liquisolid tablets at: (A) pH 1.2 using 0.1N HCl and (B) pH 6.4–6.6 using distilled water.

**Fig. 4.**
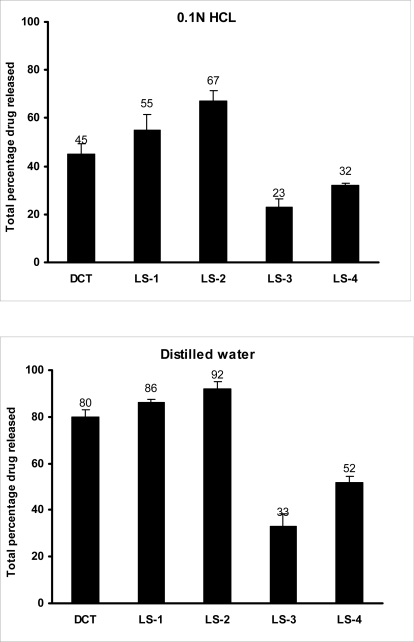
Total amount released of furosemide from conventional and liquisolid tablets using different dissolution media, for compositions refer to Table 1.

Dissolution profiles of furosemide batches in water (pH 6.4–6.6) and 0.1N HCL (pH 1.2) are presented as the percentage drug released versus time plots (Figure 3A and B). The percentage drug released after 10 minutes (DR_10min_) and half life values T_50%_ (i.e. time required to dissolve 50% of the drug) were calculated and are collected in Table 4. The total percentages of drug dissolved at the end of the dissolution experiment are presented as histograms in Figure 4.

In the acidic medium (pH 1.2), liquisolid formulations prepared with Synperonic® PE/L 81 as the liquid vehicle showed higher drug dissolution, compared to CDT and liquisolid containing PEG 400 (Figure 3A). Such formulations showed DR_10min_ of 34, 40 and 12% for LS-1, LS-2 and CDT, respectively. Meantime, T_50%_ was 50 and 20 minutes for LS-1 and LS-2, respectively, while that for CDT was out of the range of the experiment time. For liquisolid compacts prepared using PEG 400, DR_10min_ were 0.3 and 1.2% for LS-3 and LS-4, respectively, with T_50%_ of more than 60 minutes. Generally, the higher liquid vehicle to drug ratio of 4:1 (LS-2 and LS-4) showed a significant (P < 0.05) increase in drug dissolution compared to the lower liquid vehicle:furosemide ratio of 2:1 (LS-1 and LS-3). The high amount of the liquid vehicle in the higher ratio may have resulted in more of furosemide present in the molecular dispersion state or more drug solubilised in the liquid vehicle as will be explained by the DSC data (See Section 3.6). Furthermore, this could be explained on the basis that after tablet disintegrated, the liquisolid primary particles suspended in the dissolution medium partly in the molecular dispersion state, therefore the surface area of drug available for dissolution is much greater in LS-2 and LS-4 (more wetted with the liquid vehicle; giving rapid dissolution due to reduced lipophilicity of the particles) to that of furosemide particles of conventional tablets; this explanation is in agreement with Spireas and Sadu [10]. The unexpected lower dissolution of liquisolid compacts prepared with PEG 400 could be attributed to the fact that furosemide was more soluble in Synperonic® PE/L 81 (See solubility section, 3.1) than in PEG400. If the drug is more soluble in one vehicle compared to another, the more solute molecule will be in the molecular dispersion state in the former vehicle. Consequently, the more drug dissolved in Synperonic® PE/L 81 will be adsorbed on coating material (fumed silica) thus exposing more drug to the dissolution media and higher dissolution occur (Figure 3). For the remaining un-dissolved drug particles, mixing with Synperonic® PE/L 81, being a non-ionic surfactant, may result in a greater wetting and reduction in the interfacial tension between the drug particles and the dissolution media leading to increased drug particle surface area in contact with dissolution media and subsequent improvement in drug solubility. Other possible explanation for the lower dissolution from liquisolid compacts with PEG 400 could be due to the poor disintegration and the high tablet hardness as explained earlier (See section 3.4). The interaction between the drug with the vehicle could be a possibility for these results, therefore characterisation of the systems was performed using thermal analysis and infrared spectroscopy (See below).

On performing the dissolution study using distilled water (Fig. 3B), all formulations displayed higher drug dissolution compared to that at 0.1N HCL. In case of formulations using Synperonic® PE/L 81 as a liquid vehicle (LS-1 and LS-2), there was a significant (P < 0.05) difference in percentage drug released compared to liquisolid compacts prepared with PEG400 (LS-3 and LS-4). In addition, the total amount of the drug dissolved from LS-1 and LS-2 at the end of the dissolution was significantly high (P < 0.05) compared to that from conventional tablets, CDT (Fig. 3B). The DR_10min_ and T_50%_ values for LS-1, LS-2 and CDT in distilled water were higher than those in the acidic medium. For drug dissolution from PEG 400 formulations in distilled water still inferior to that of Synperonic PE/L 81 liquisolid compacts as well as conventional tablets. Again, compacts with higher vehicle:drug ratio of 4:1, showed a significant (P < 0.05) increase in drug dissolution. The higher drug dissolution at the higher pH value could be due to the fact that furosemide is a weak acidic drug with pK_a_ value of 3.9 [20]. In distilled water, where pH value (ranged from 6.4 to 6.6) is higher than drugs’ pK_a_, the carboxylic group of furosemide will be highly ionised and consequently drug solubility increases.

### Differential Scanning Calorimetry (DSC)

**Fig. 5.**
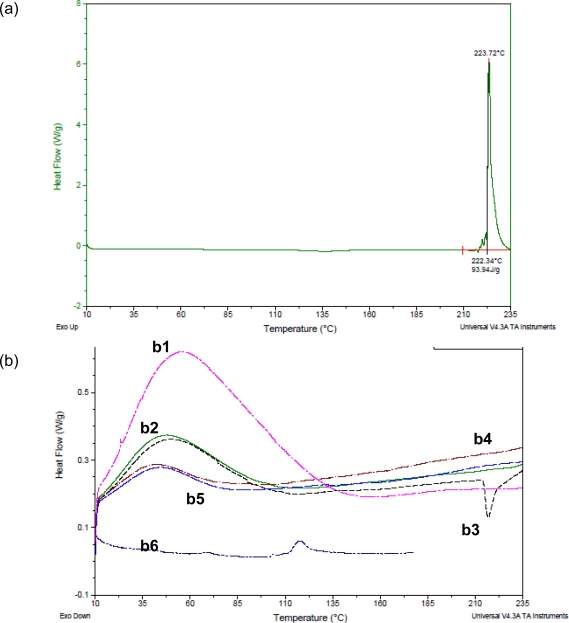
DSC thermograms of (a) furosemide and (b) pure excipients and furosemide whithin conventional and liquisolid formulations where b1 (pink line) is potato starch, b2 is Avicel® PH 101 (green line), b3 (black line) is conventional powder, b4 (dark red line) is LS-1, b5 (faint blue line) is LS-3 and b6 (dark blue line) is fumed silica. For compositions refer to Table 1.

In a trial to explain the dissolution data, thermal analysis was conducted to establish the existence of any possible interaction between the drug and tablet excipients. Thermograms of furosemide test systems are shown in Figure 5.

Furosemide alone exhibited a characteristic, sharp peak at 223.7°C, which indicates its crystalline nature. It is known that disappearance or shifting of endo-or exothermic peaks is an indication of change in crystalline structure of the drug. In conventional formulation, CDT, there was shifting in the furosemide characterstic peak to lower temperature (217°C) with reduction in enthalpy indicating reduced drug crystallinity. In the case of LS-1 (Figure 5) and the same for LS-2 (data not shown), there were complete disappearance of furosemide characteristic peak, indicating complete loss of crystallinity or amorphisation of the drug that could explain the improved drug dissolution over conventional type. For PEG 400 liquisolid compacts (Figure 5), furosemide characteristic peak was not found indicating amorphisation, with a slight shift in the endothermic peak of Avicel® PH 101 with reduced enthalpy, indicating complete solubilisation and/or dispersion of the drug in the liquid vehicles forming uniform dispersion in the carrier with a possible formation of a complex. The formation of this complex could be the reason for poor dissolution of furosemide from these formulations. Therefore, FT-IR study was conducted to investigate the possible interaction between the drug and the other components of the tablets.

### Fourier Transform Infrared Spectroscopy

**Fig. 6.**
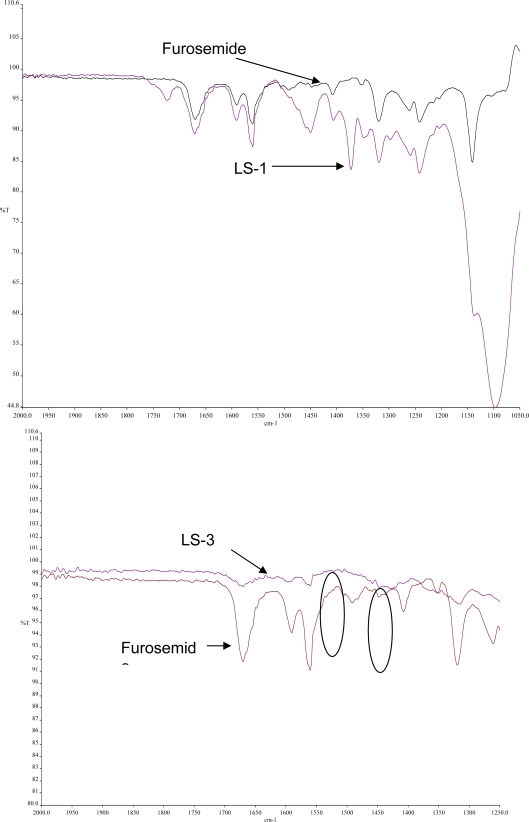
FT-IR spectra of furosemide and liquisolid formulations: LS-1 and LS-3. For compositions refer to Table 1.

FT-IR was performed for the pure drug and liquisolid powders to detect any sign of interaction which would be reflected by a change in the position or disappearance of any characteristic stretching vibration of the compound. From the infrared spectrum of pure furosemide, absorption bands are observed at 3340 and 3260 cm^−1^ and sharp bands are observed at 1665 and 1560 cm^−1^ (Figure 6). The 3340 cm^−1^ band is assigned to the NH_2_ stretching vibration of Ar---NHCH_2_ and the 3260 cm^−1^ band is assigned to stretching vibration of SO_2_NH_2_ and the 1665 cm^−1^ band, which appears at such high frequency region, is assigned to the bending vibration of amino group. The 1560 cm^−1^ band is due to the assymmetric stretching vibration of the carbonyl group and the 1318 cm^−1^ band is assigned to the assymmetric stretching vibration of the sulfonyl group in the furosemide structure [21–22].

In the case of liquisolid formulations containing Synperonic® PE/L 81 (LS-1 and LS-2), all peaks of the drug still shown in the spectrum indicating that there was no interaction between the drug and the vehicle and the drug was molecularly dispersed in the liquid vehicle, confirming the DSC data. Nevertheless, for liquisolid formulations containing PEG 400 (LS-3), it is clear from the spectrum that there was a change in the spectrum compared to that of pure furosemide (decreased intensity of most peaks in addition to disappearance of some characteristic bands of the drug, e.g. peak corresponds to the stretching vibration of the sulfonyl group at 1318 cm^−1^). Also, the peaks for imino- and sulfonylamide vibrations were disappeared and replaced by a broad peak. These results would strengthen our previous assumption that interaction such as association between functional groups of furosemide and PEG 400 at the molecular level had occurred resulting in formation of a complex that was less soluble than the parent compound. The association between furosemide and PEG 400 is expected to be most probably between the imino group and the sulfonylamide group of furosemide and the terminal hydroxyl groups of PEG400.

## Conclusion

From the conducted work it is possible to conclude that, based on the nature of the drug and the non-volatile liquid vehicle used, liquisolid technique has the capability to increase furosemide intrinsic solubility. Liquisolid tablets containing Synperonic® PE/L 81 as a new liquid vehicle exhibited greater dissolution due to the physical properties of this liquid vehicle which led to increased wetting properties and solubility of the drug; demonstrating higher drug release than those of conventionally made tablets (which failed as well friability test and could not withstand). PEG 400 as a liquid vehicle failed to improve furosemide dissolution owing to lower solubility of the drug in PEG 400 compared to Synperonic® PE/L 81 and possible drug-PEG 400 interaction as revealed by DSC and FT-IR data. Since drug dissolution is the rate limiting step in oral drug absorption of non-polar molecules, liquisolid compacts prepared with Synperonic® PE/L 81 might present substantial in vivo superiority over conventional directly compacted counterpart. Caprol® PGE 860 was not a good choice of liquid vehicles to prepare furosemide liquisolid tablets. There is no single liquid vehicle which is suitable for all poorly water soluble drugs to formulate liquisoid compacts. Accordingly, choosing a suitable liquid vehicle, depending on its properties e.g. viscosity, for a particular drug is important to prepare a successful liquisolid tablets.

## Experimental

### Materials

Furosemide was provided by Sigma-Aldrich Company Ltd (Gillingham UK). Microcrystalline cellulose (Avicel® PH101) (FMC Corp., Philadelphia, USA), colloidal silicon dioxide (Cab-o-sil® M-5, fumed silica) (Cabot Corporation, Rheinfelden, Germany), potato starch (BDH laboratory supplies, Poole, England), 1,2,3-propanetriol, homopolymer, (9Z)-9-octadecenoate (Caprol® PGE-860) (Abitec Corporation, Columbus, USA), Synperonic® PE/L 81 (polyoxyethylene-polyoxypropylene-polyoxyethylene block copolymer) (ICI surfactants, Everberg, Belgium) and polyethylene glycol 400 (PEG 400) (BDH laboratory supplies, Poole England) were used. Other reagents were of analytical grade.

### Theoretical aspects for designing the liquisolid systems

The amounts of excipients (carrier and coating materials) used to prepare liquisolid compacts depend on the flowable liquid retention potential values (Φ-value) and the liquid loading factors (L_f_), Equation 1. The Φ-value of a powder is the maximum amount of a given non-volatile liquid that can be retained inside powder bulk (w/w) while maintaining acceptable flowability. Whereas, L_f_ is the mass ratio (w/w) of the liquid medication to the carrier powder in the liquisolid formulation, and it is given by Equation 2. Therefore, in order to calculate the required weight of excipients, we need to determine the liquid retention potential value for both carrier (ΦCA-value) and coating (ΦCO-value) materials for each non-volatile liquid vehicle used, these values are constant for the given vehicle/powder system. Knowing the carrier:coating ratio (R), which is 20:1 in this study, liquid loading factor (L_f_) can be calculated by the following equation:

Eq. 1. $${\text{L}}_{\text{f}}=\Phi \text{CA}+\Phi \text{C}0\cdot \frac{1}{R}$$

Once liquid loading factors were obtained for such non-volatile liquid vehicle used in this study, the optimum weight of the carrier (Q), required for the respective vehicle could be calculated by rearranging the Equation (2).

Eq. 2. $${\text{L}}_{\text{f}}=\frac{\text{W}}{\text{Q}}$$

Where: W is the weight of the liquid medication (the drug + non-volatile liquid vehicle) and Q is the weight of the carrier [7, 9]. The weights of the liquid medications (the drug + non volatile liquid vehicle) were calculated at drug:vehicle ratios of 1:2 and 1:4. Once the values for Q were obtained for the respective vehicle, the optimum weight of the coating material (q) could also be obtained (Equation 3).

Eq. 3. $$\text{R}=\frac{\text{Q}}{\text{q}}$$

Where: Q and q are the weight of the carrier and coating material, respectively.

According to the theories of Spireas and his co-workers [4, 8, 21], the carrier and coating powder materials can retain only certain amount of the liquid vehicle while maintaining acceptable flow and compaction properties. The amounts of the drug, carrier and coating materials required to produce sixty tablets were then calculated and powders were prepared. The outline of the constituents of each of the formulation prepared from the previously mentioned variables are demonstrated in Table 1.

### Solubility Studies

To determine the best non-volatile liquid vehicle for dissolving or suspending furosemide in liquid medication, the solubility of furosemide in Synperonic® PE/L 81, PEG 400, Caprol® PGE 860 and water was determined. Excess furosemide was added to 5ml of each liquid vehicle and was shaken in a shaker (Stuart Company Ltd, England) for 72 hours at 25°C under constant vibration. After this period, aliquots were taken, filtered through 0.45μm Millipore filter, diluted with distilled water and analysed by UV-Vis Spectrophotometer (CE292, Cecil Instruments, Cambridge, UK) at 228 nm. Three determinations were carried out for each sample to calculate the solubility of furosemide in each vehicle.

### Determination of the viscosity of different liquid vehicles

Viscosities of the non-volatile liquid vehicles (Caprol® PGE-860, Synperonic® PE/L 81, PEG 400) were determined at 25°C using Ostwald U-tube (D22596) viscometer. The rate of flow of the liquid via the capillary was measured under the effect of gravity.

### Determination of angle of slide for Avicel®PH 101 (carrier material) and fumed silica (coating material)

Powder flowability is of critical importance in production of solid pharmaceutical dosage forms in order to get a uniform feed as well as reproducible filling of tablet dies, otherwise, high dose variation will occur. In order to ensure the flow properties of the liquisolid systems to be compacted into tablet, the angle of slide for Avicel®PH 101 (carrier material) and fumed silica (coating material) were measured. Exactly weighted 10 g of the carrier or coating material were placed at one end of a metal plate with a polished surface. The plate was gradually raised until the plate made an angle (θ, angle of slide) with the horizontal plane at which the powder was about to slide over the polished surface [11]. An angle of slide of 33º corresponded to optimum flow [11].

### Determination of flowable liquid retention potential for Avicel® PH 101 (Φ_CA_-value) and fumed silica (Φ_CO_-value)

An increasing amount of the non-volatile liquid vehicles (Synperonic® PE/L 81 or Caprol® PGE 860) were added to 10 g of Avicel® PH 101 or silica powder and mixed using pestle and a mortar to give powder admixtures. The carrier and coating materials adsorbed the liquid vehicle resulting in a change in material flow properties compared to pure powder of Avicel® PH 101 or silica powder previously measured (See section 2.5). At each concentration of the non-volatile liquid vehicle, the angle of slide was determined as stated previously (section 2.5). The corresponding flowable liquid retention potentials were calculated using the following equation:

Eq. 4. $$\Phi -\text{value}=\frac{\text{weight}\;\text{of}\;\text{liquid}}{\text{weight}\;\text{of}\;\text{solid}}$$

Then, the obtained Φ-values were plotted against the corresponding angle of slides. The Φ-value which corresponded to an angle of slide of 33° represented the flowable liquid retention potentials of powder admixture [23]. In cases where the Φ-value did not correspond to 33°, the highest Φ-value reached was chosen as the flowable liquid retention potential. Figures 1 and 2 show the results. The ΦCA-value for Avicel® PH 101 with PEG 400 and the ΦCO-value for fumed silica with PEG400 were reported to be 0.005 and 3.26, respectively [4].

### Preparation and mixing of powders for conventional and liquisolid tablets

A conventional formulation of furosemide, (denoted as CDT; conventional) was directly compacted into tablets, each containing 20 mg drug. In addition, each tablet contained 200 mg Avicel® PH 101, 10 mg fumed silica and 10 mg potato starch (as a disintegrant); a batch of 60 tablets was mixed in a turbular mixer (Erweka, Germany) and compacted using a 10mm flat-faced punch and die set tabletting machine (type F3, Manesty Machine Ltd., Liverpool, UK).

In the case of liquisolid formulations, from the obtained results of optimum ΦCA-and ΦCO-values (optimum flowable liquid retention values for the carrier and coating materials, respectively) with each liquid vehicle, the optimum liquid load factors (L_f_) were calculated using Equation (1) with carrier:coat ratio (R) of 20. Then, the amount of carrier (Q) and coating (q) materials were calculated using Equations 2 and 3 at drug:vehicle ratios of 1:2 and 1:4. Several liquisolid systems of furosemide (LS-1 to LS-6; Table 1) were prepared in 60 tablet batches of 20 mg strength each. The calculated amounts of the carrier (Q) and coating (q) materials at each liquid medication (W) are presented in Table 2.

Accordingly, the liquisolid formulations were prepared as follows: the drug was suspended in the liquid vehicle in a mortar using pestle, then the calculated amount of the carrier material (Avicel® PH 101) was added with continuous mixing till homogenous wet mix is obtained. The coating material (fumed silica) was then added to the mix with gentle mixing (the powder admixture re-establish the dry powder consistency). Finally, each liquisolid formulation was blended with 5%w/w of a disintegrating agent (potato starch).

### Determination of flow properties of prepared powder admixtures:

Before compaction liquisolid powder admixture into compacts it was necessary to study the flowability of these systems. Flowability was assessed from Carr’s compressibility Index (CI%). The CI was calculated from the poured (bulk) and tapped densities. Tapped density was measured by tapping fixed weight of the sample into 100 ml measuring cylinder several times using a tap density apparatus (J. Engelsmann A.G., Ludwigshafen, Germany) till a constant volume is obtained, where the powder is considered to reach to its most stable arrangement. Carr’s compressibility index was then calculated using the following Equation [24]:

Eq. 4. $$\text{CI}\%=\frac{\text{tapped}\;\text{density}-\text{bulk}\;\text{density}}{\text{tapped}\;\text{density}}\cdot 100$$

The smaller the value of the CI%, the superior the flow properties of the powder [11].

### Compaction of the prepared powder admixtures

The liquisolid powder admixtures (LS-1-LS-4) were compacted into tablets (60-tablet batches) containing 20 mg furosemide per tablet using the previously mentioned tabletting machine. The compression force that applied was sufficient to produce acceptable tablet hardness. Formulations LS-5 and LS-6 (with Caprol® PGE 860 as a liquid vehicle) were not compactible.

### Evaluation of furosemide conventional and liquisolid tablets:

Tablets were evaluated by performing quality control tests for uniformity of drug content, friability, disintegration, hardness as well as dissolution test. All tests were carried out in triplicates and according to compendial specifications [19].

1. Drug content uniformity of furosemide was determined by collecting a sample of 10 tablets from each batch followed by a determination of the drug concentration in each tablet spectrophotometrically at 228 nm. The average drug content is calculated and the percentage drug content of the individual tablet should fall within specified limits in terms of percentage deviation from the mean.
2. Friability was determined using Copley friabilator (FRV 1000, Copley Scientific, UK), the percentage loss in tablet weight before and after 100 revolution of 20 tablets were calculated and taken as a measure for friability.
3. Disintegration time; the time necessary to disintegrate 6 tablets of each tablet formulation was determined using disintegration tester (Manesty Machine Ltd., Liverpool, UK).
4. Hardness; It is a measure of the mechanical strength of a tablet using hardness tester (Model 2E/205, Schleuniger & Co., Switzerland). The mechanical strength of a tablet is associated with the resistance of a tablet to fracture or attrition.
5. In vitro dissolution studies; The USP dissolution apparatus II (Caleva Ltd. Dorset, UK) was used with 900 ml, to ensure sink conditions, of distilled water (pH range of 6.4–6.6) and 0.1N HCL (pH 1.2) at 37±0.5°C; the apparatus was run at 100 rpm. Samples of the dissolution medium were withdrawn at a specified time intervals and compensated by fresh dissolution medium. Samples were properly diluted and furosemide concentrations were analysed spectrophotometrically at 228nm. The cumulative percentage drug released at each time interval was calculated and plotted against time.

### Differential scanning calorimetry (DSC)

DSC thermograms of furosemide, Avicel® PH101, fumed silica, potato starch, conventional and liquisolid formulations were generated with a DSC Refrigerated Cooling System (Model Q1000, TA Instruments, UK). Tested samples were weighed and analysed hermetically in sealed aluminium pans. The instrument was calibrated with sapphire and indium before running the samples. The samples were examined from 10°C to 235°C at scanning rate of 10°C/minute.

### Fourier Transferom Infrared Spectroscopy

Infrared spectra of the samples (furosemide and liquisolid formulations) were obtained, using Perkin Elmer FT-IR system Spectrum BX series (Beaconsfield, Buckinghamshire, UK), in the frequency range of 4000–550 cm^−1^ at 4 cm^−1^ resolution. The technique utilised very small amount of each sample which directly loaded into the system. Spectrum BX series software version 2.19 was used to determine peak positions.

### Statistical analysis

One-way ANOVA and Independent-samples T-test were applied if the variances in the groups are equal. If the variances are significantly different, Mann-Whitney Test was used. Results are statistically significant when P <0.05.

## Figures and Tables

**Tab. 1.** Summary of the prepared liquisolid formulations.

**Liquisolid Formulation code**	**Liquid vehicle**	**Drug:vehicle ratio**
LS-1	Synperonic® PE/L 81	1:2
LS-2	Synperonic® PE/L 81	1:4
LS-3	PEG 400	1:2
LS-4	PEG 400	1:4
LS-5	Caprol® PGE 860	1:2
LS-6	Caprol® PGE 860	1:4

**Tab. 2.** Composition of different furosemide liquisolid formulations prepared using different liquid vehicles according to theoretical model by Spireas and Bolton [4].

**Batch code^a^**	**Drug:liquid vehicle ratio**	**W (mg)**	**L_f_**	**Avicel® PH 101 (mg) (Q=W/L_f_)**	**Fumed silica (mg) (q=Q/R)**	**Potato starch (mg)**	**Unite dose weight (mg)**
LS-1	1:2	60	0.274	218.98	10.95	14.50	304.40
LS-2	1:4	100	0.274	364.96	18.25	24.16	507.40
LS-3	1:2	60	0.168	357.10	17.86	21.75	456.71
LS-4	1:4	100	0.168	595.20	29.76	36.25	761.21
LS-5	1:2	60	0.837	71.64	3.51	6.76	141.91
LS-6	1:4	100	0.837	119.40	5.90	11.27	236.57

W … weight of liquid medication (drug + liquid vehicle); L_f_ … liquid load factor; Q … weight of carrier material; q … weight of coating material; R … carrier:coat ratio which equals to 20:1.

a for the composition of each formula refer to Table 1.

**Tab. 3.** Characteristics of the formulated furosemide tablets.

**Batch code^a^**	**Average furosemide contents (% ±SD)**	**% Friability**	**Hardness (N ±SD)**	**Disintegration Time (min ±SD)**
CDT	97.32 (± 1.7)	1.40	27.4 (± 9.64)	0.82 ± 0.34
LS-1	99.91 (± 1.5)	0.51	39.4 (± 7.99)	0.35 ± 0.16
LS-2	100.03 (± 3.7)	0.32	46.9 (± 11.6)	0.27 ± 0.11
LS-3	100.02 (± 2.8)	0.14	98.1 (± 8.19)	19.3 ± 2.1
LS-4	101.02 (± 1.7)	0.22	107.9 (± 12.7)	22.5 ± 1.6

a for the composition of each formula refer to Table 1.

**Tab. 4.** The total amount of drug released after 10 minutes (DR_10min_) and the time necessary to release 50% of the drug from different formulations at different dissolution media.

**Formula Code^a^**	**0.1N HCl (pH1.2)**		**Distilled water (pH 6.4–6.6)**	

	**DR_10min_ (% ±SD)**	**T_50%_ (min ±SD)**	**DR_10min_ (%±SD)**	**T_50%_ (min ±SD)**
DCT	24 (±2.1)	>60	57 (±3.2)	10 (±1.2)
LS-1	34 (±3.1)	50 (±3.5)	52 (±3.6)	8.0 (±1.4)
LS-2	40 (±1.9)	20 (±2.8)	64 (±2.7)	6.0 (±1.3)
LS-3	0.9 (±0.51)	>60	1.3 (±0.71)	>60
LS-4	2.2 (±0.92)	>60	2.9 (±1.1)	59 (±4.8)

a for the composition of each formula refer to Table 1.
